# The solute carrier SLC7A8 is a marker of favourable prognosis in ER-positive low proliferative invasive breast cancer

**DOI:** 10.1007/s10549-020-05586-6

**Published:** 2020-03-21

**Authors:** Rokaya El Ansari, Lutfi Alfarsi, Madeleine L. Craze, Brendah K. Masisi, Ian O. Ellis, Emad A. Rakha, Andrew R. Green

**Affiliations:** 1 Nottingham Breast Cancer Research Centre, Division of Cancer and Stem Cells, School of Medicine, University of Nottingham Biodiscovery Institute, University Park, Nottingham, NG7 2RD UK; 2 Histopathology, Nottingham University Hospitals NHS Trust, Hucknall Road, Nottingham, NG5 1PB UK; 3 Department of Pathology, Faculty of Medicine, University of Tripoli, Tripoli, Libya

**Keywords:** SLC7A8, Breast cancer, Prognosis

## Abstract

**Purpose:**

Breast cancer (BC) is a heterogeneous disease consisting of various subtypes, with different prognostic and therapeutic outcomes. The amino acid transporter, SLC7A8, is overexpressed in oestrogen receptor-positive BC. However, the consequence of this overexpression, in terms of disease prognosis, is still obscure. This study aimed to evaluate the biological and prognostic value of SLC7A8 in BC with emphasis on the intrinsic molecular subtypes.

**Methods:**

SLC7A8 was assessed at the genomic, using METABRIC data (*n* = 1980), and proteomic, using immunohistochemistry and TMA (*n* = 1562), levels in well-characterised primary BC cohorts. SLC7A8 expression was examined with clinicopathological parameters, molecular subtypes, and patient outcome.

**Results:**

*SLC7A8* mRNA and SLC7A8 protein expression were strongly associated with good prognostic features, including small tumour size, low tumour grade, and good Nottingham Prognostic Index (NPI) (all *P* < 0.05). Expression of *SLC7A8* mRNA was higher in luminal tumours compared to other subtypes (*P* < 0.001). High expression of *SLC7A8* mRNA and SLC7A8 protein was associated with good patient outcome (*P* ≤ 0.001) but only in the low proliferative ER+/luminal A tumours (*P* = 0.01). In multivariate analysis, *SLC7A8* mRNA and SLC7A8 protein were independent factors for longer breast cancer specific survival (*P* = 0.01 and *P* = 0.03), respectively.

**Conclusion:**

SLC7A8 appears to play a role in BC and is a marker for favourable prognosis in the most predominant, ER+ low proliferative/luminal A, BC subtype. Functional assessment is necessary to reveal the specific role played by SLC7A8 in ER+ BC.

**Electronic supplementary material:**

The online version of this article (10.1007/s10549-020-05586-6) contains supplementary material, which is available to authorised users.

## Introduction

Many cancer cells alter their metabolism to provide energy and cellular building blocks required for their rapid proliferation. Amino acids, particularly glutamine and leucine, are essential for cancer cell growth, as they are critical for controlling protein translation and driving cell cycle progression through regulation of the mammalian target of rapamycin complex1 (mTORC1) pathway [1, 2]. This pivotal need for intracellular amino acids is reflected in the increased expression of amino acid transport systems in the majority of cancers, which is regulated by various transcription factors such as c-MYC, hormone receptors, and nutrient starvation responses [3–5].

Solute Carrier Family 7 Member 8 (SLC7A8) and Member 5 (SLC7A5) are sodium-independent amino acid exchangers (antiports), which transport small and large neutral amino acids, such as alanine, serine, threonine, cysteine, phenylalanine, tyrosine, leucine, and glutamine [6]. Both solute carriers require heterodimerisation with the heavy chain of SLC3A2 for their proper localisation in the plasma membrane [7–9].

Although the function of SLC7A8 and SLC7A5 are similar, the former displays relatively lower affinity for its substrates, glutamine, and serine. SLC7A5 has been extensively studied in a variety of cancers and it is regulated by the oncogene c-MYC [10–13]. We have previously described the potential utility of SLC7A5 as a poor prognostic factor for the highly proliferative breast cancer (BC) subtypes [14]. However, there is limited information whether SLC7A8 plays an equal role in BC. Previous studies showed that SLC7A8 is upregulated in ER+BC and it is controlled by oestrogen [4, 15]. Luo et al. also identified SLC7A8 as a novel progesterone target gene in uterine leiomyoma cells [16]. To our knowledge, the prognostic impact of SLC7A8 has not been studied.

In this study, we aimed to assess *SLC7A8* gene copy number (CN) and mRNA expression, alongside SLC7A8 protein expression in large and well-characterised cohorts of BC to determine its clinicopathological and prognostic value with emphasis on the different molecular classes.

## Material and methods

### *SLC7A8* genomic profiling

*SLC7A8* gene copy number and gene expression were evaluated using the Molecular Taxonomy of Breast Cancer International Consortium (METABRIC) cohort of invasive BC (*n* = 1980) [17]. In this study, DNA/RNA was isolated from fresh frozen samples and transcriptional profiling was acquired using the Illumina HT-12v3 platforms. Data were pre-processed and normalised as described previously [17]. Patients involved in the study who were Oestrogen Receptor-negative (ER-) and Lymph Node (LN)-positive received adjuvant chemotherapy, while ER+ and/or LN− patients did not receive adjuvant chemotherapy. Dichotomisation of *SLC7A8* mRNA was achieved using X-tile (version 3.6.1, Yale University, USA), based on prediction of Breast Cancer Specific Survival (BCSS). *SLC7A8* mRNA expression was associated with clinicopathological parameters, molecular subtypes and patient outcome.

The online dataset, Breast Cancer Gene-Expression Miner v4.0 (https://bcgenex.centregauducheau.fr), was used for external validation of *SLC7A8* mRNA expression.

### SLC7A8 protein expression

Immunhistochemistry for SLC7A8 was performed using a well-characterised cohort of early stage primary operable invasive BC patients aged ≤ 70 years. Patients presented at Nottingham City Hospital between 1989 and 2006. Patients were managed based on a uniform protocol. Clinical history, tumour characteristics, information on therapy, and outcomes are prospectively maintained. Outcome data included development and time to distant metastasis (DM) and BCSS.

Supplementary Table 1 summarises the clinicopathological parameters for the Nottingham and METABRIC series.

### Western blotting

The specificity of anti-SLC7A8 primary antibody (HPA051950, Sigma-Aldrich, UK) was validated using Western blotting in BC lysates (American Type Culture Collection; Rockville, MD, USA) as previously described [18]. A single band for SLC7A8 was visualised at the correct predicted size (~ 58 KDa) (Supplementary Fig. 1).

### Tissue arrays and Immunohistochemistry (IHC)

Tumour samples, 0.6 mm cores, were arrayed as previously described [14, 19]. Immunohistochemical staining was performed on 4 μm TMA sections using Novolink polymer detection system (Leica Biosystems, RE7150-K) as per the manufacturer’s instructions.

Stained TMA sections were scanned using high resolution digital images (NanoZoomer; Hamamatsu Photonics, Welwyn Garden City, UK), at × 20 magnification. Modified histochemical score (H-score) was applied to evaluate SLC7A8 immunostaining. This includes a semi-quantitative assessment of both the percentage and the intensity of stained cells [20]. Staining intensity was graded as: 0, negative; 1, weak; 2, medium; 3, strong and the percentage of the positively stained tumour cells was estimated subjectively. The final H-score was calculated multiplying the intensity (0–3) by the percentage of positive cells (0–100), producing a total range of 0–300. Dichotomisation of SLC7A8 protein expression was determined using X-tile software in predicting BCSS.

Immunhistochemical staining and dichotomisation of the other biomarkers included in this study were as per previous publications [14, 18–22]. ER and PgR positivity was defined as ≥ 1% staining. Immunoreactivity of HER2 was scored using standard HercepTest guidelines (Dako). Chromogenic in situ Hybridisation (CISH) was used to quantify HER2 gene amplification in borderline cases using the HER2 FISH pharmDx™ plus HER2 CISH pharmDx™ kit (Dako) and was assessed according to the American Society of Clinical Oncology guidelines. BC molecular subtypes were defined, based on tumour IHC profile and the Elston-Ellis [23] mitotic score as: ER+/HER2- Low Proliferation (mitotic score 1), ER+/HER2− high Proliferation (mitotic score 2 and 3), HER2− positive class: HER2+ regardless of ER status, Triple Negative (TN): ER−, PgR−, and HER2− [24].

### Statistical analysis

SPSS 24.0 statistical software (SPSS Inc., Chicago, IL, USA) was applied for statistical analysis. The Chi-square test was carried out for inter-relationships between categorical variables. One-way ANOVA with post hoc Tukey multiple comparison test and Pearson’s correlation coefficient was performed to analyse the association between continuous variables. Survival curves were examined by Kaplan–Meier with Log Rank test. Cox’s proportional hazard method was performed for multivariate analysis to identify the independent prognostic/predictive factors. *P* values were adjusted using Bonferroni correction for multiple testing, whenever applicable. A *P* value < 0.05 was considered significant. The study endpoints were 10-year BCSS or distant metastasis free survival (DMFS). This study complied with reporting recommendations for tumour marker prognostic studies (REMARK) criteria [25].

This study was approved by the Nottingham Research Ethics Committee 2 under the title ‘Development of a molecular genetic classification of breast cancer’ and the North West—Greater Manchester Central Research Ethics Committee under the title ‘Nottingham Health Science Biobank (NHSB)’ reference number 15/NW/0685.

## Results

### SLC7A8 in breast cancer

**Fig. 1:**
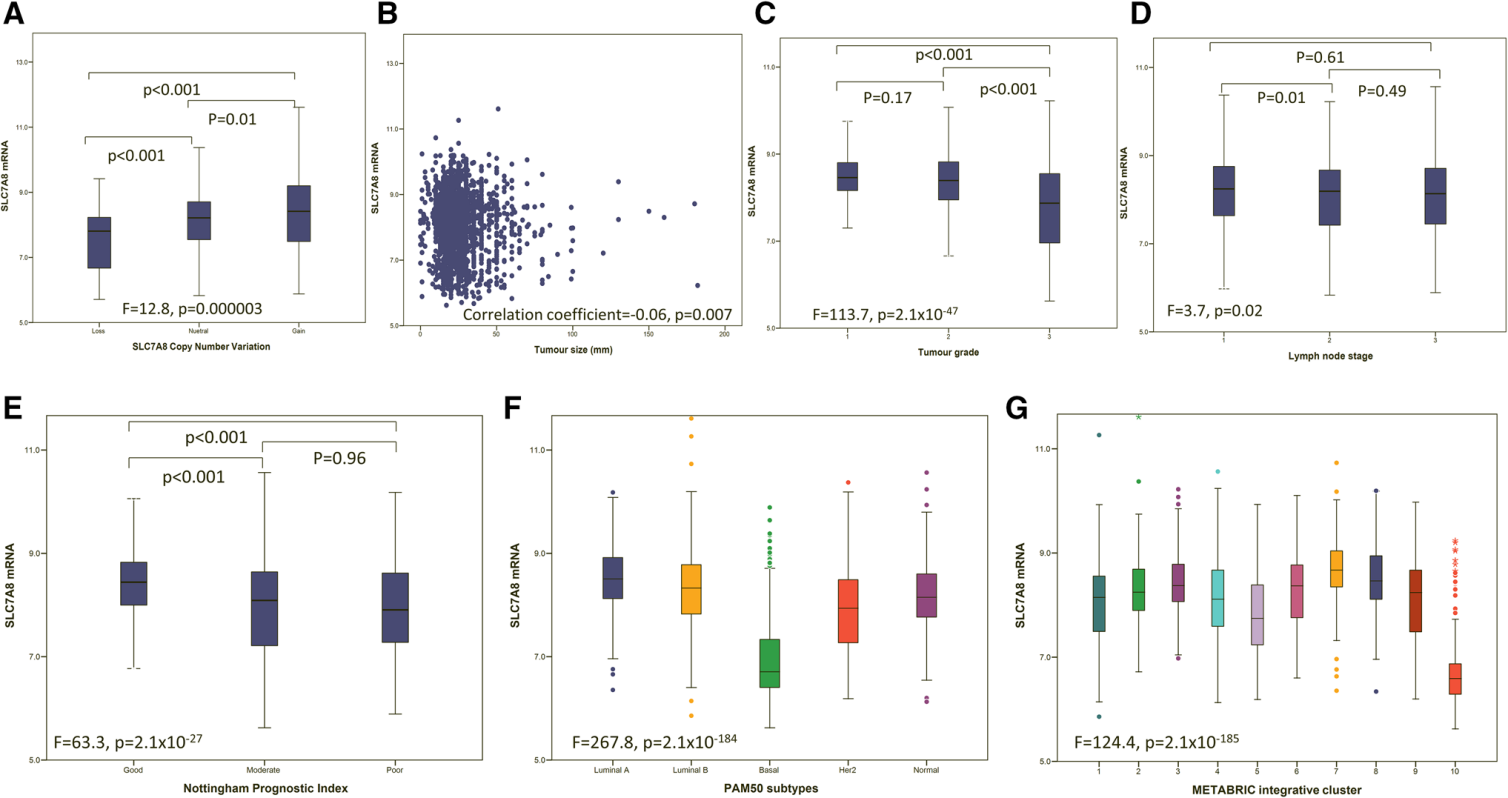
*SLC7A8* mRNA expression and its association with copy number aberrations, clinicopathological parameters and molecular subtypes: **a** SLC7A8 and copy number aberrations, **b***SLC7A8* and tumour size, **c***SLC7A8* and tumour grade, **d***SLC7A8* and lymph node stage, **e***SLC7A8* and NPI, **f***SLC7A8* and PAM50 subtypes, **g***SLC7A8* and METABRIC Integrative Clusters. Pearson correlation was used for two variables and One-way ANOVA with post hoc tukey test for more than two variables

High *SLC7A8* mRNA expression was observed in almost two-third (67%) of the METABRIC BC cases. A total of 90/1,980 (4.5%) of cases showed *SLC7A8* copy number (CN) gain, whereas 45/1,980 (2.3%) cases showed a CN loss. A significant association was observed between *SLC7A8* copy number variation (CNV) and *SLC7A8* mRNA expression (*P* < 0.0001, Fig. 1a). There was a positive association between SLC7A8 CN gain and CN gain of the tumour suppressor gene, TP53 (*P* < 0.0001, Supplementary Table 2).

**Fig. 2:**
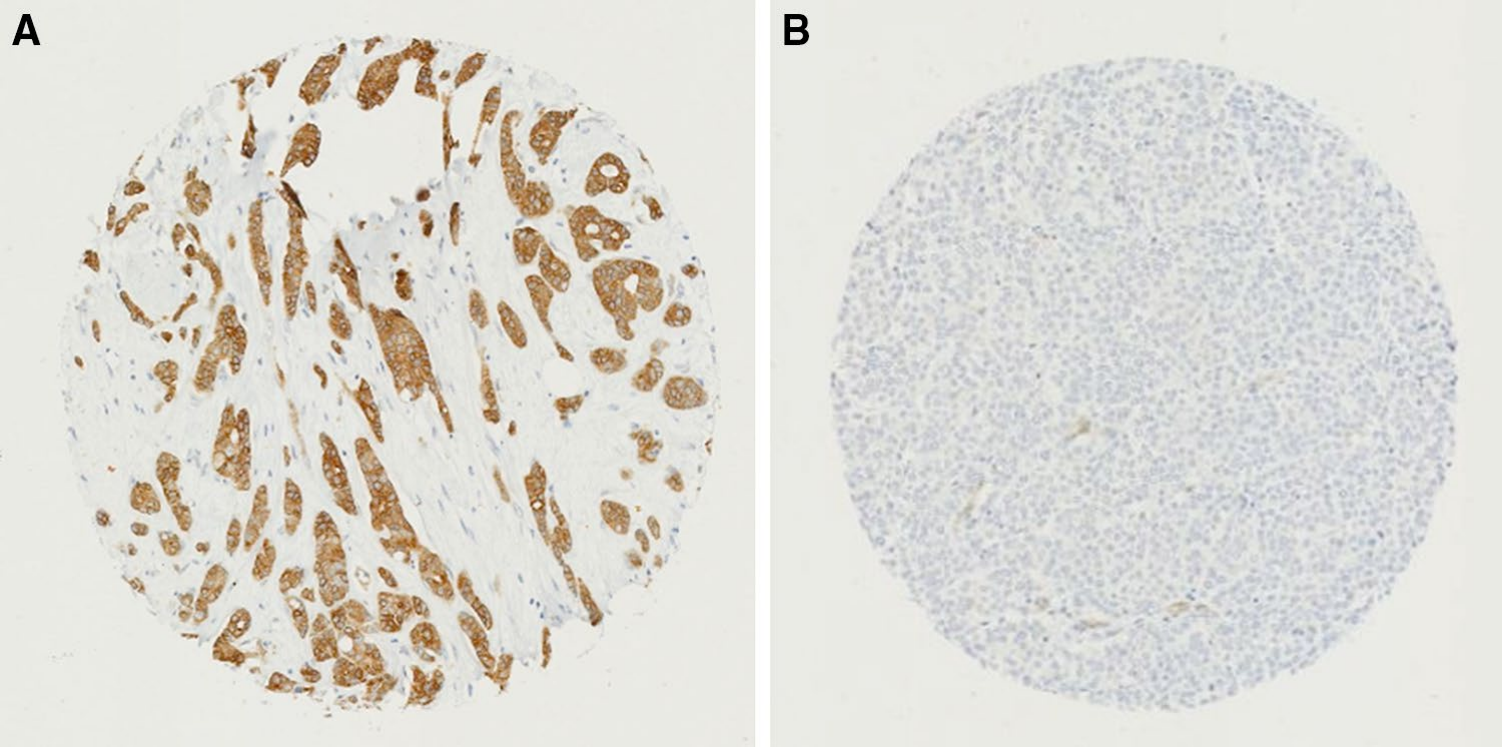
SLC7A8 protein expression in invasive breast cancer cores. **a** Positive IHC expression, **b** negative IHC expression

SLC7A8 protein expression was observed, predominantly in the cytoplasm of invasive BC cells, with expression levels varying from absent to high (Fig. 2a and b). Positive SLC7A8 protein expression (> 20 H-score) was observed in 177/1560 (11%) of cases.

### SLC7A8 and clinicopathological parameters

High *SLC7A8* mRNA expression was significantly associated with good prognostic parameters, including smaller tumour size (Fig. 1b, *P* = 0.007), lower tumour grade (Fig. 1c, *P* < 0.001), and good Nottingham Prognostic Index (NPI) (Fig. 1e, *P* < 0.001). These associations were confirmed using the Breast Cancer Gene-Expression Miner (Supplementary Fig. 2A–2C).

Similar associations were observed with SLC7A8 protein expression. Table 1 summarises the observed findings between high SLC7A8 protein and good prognostic factors, including small tumour size (*P* = 0.03), low tumour grade (*P* < 0.001), and good NPI (*P* < 0.001).

**Table 1:** Clinicopathological associations of the SLC7A8 protein expression in breast cancer

Parameter	SLC7A8 protein		*χ*^2^ (*P *value)	Adjusted *P *value
	Low *n* (%)	High *n* (%)		
Tumour size				
< 2 cm	756 (87.1)	112 (12.9)	7.03 (0.008)	**0.03**
≥ 2 cm	623 (91.3)	59 (8.7)		
Tumour grade				
1	182 (82.7)	38 (17.3)	20.62 (0.00003)	**0.0002**
2	491 (86.7)	75 (13.3)		
3	704 (92.4)	58 (7.6)		
Lymph node stage				
1	840 (87.9)	116 (12.1)	4.84 (0.08)	0.16
2	400 (89.7)	46 (10.3)		
3	136 (93.8)	9 (6.2)		
Nottingham Prognostic Index				
Good	397 (83.8)	77 (16.2)	20.09 (0.00004)	**0.0002**
Moderate	746 (90.6)	77 (9.4)		
Poor	234 (93.2)	17 (6.8)		
IHC subtypes				
ER+/HER2− low proliferation	706 (87.7)	99 (12.3)		
ER+/HER2− high proliferation	230 (90.9)	23 (9.1)	5.67	
Triple negative	207 (88.5)	27 (11.5)	(0.128)	0.24
HER2+	145 (93.5)	10 (6.5)		
Histological type				
Ductal (including mixed)	1217 (89.6)	142 (10.4)		
Lobular	88 (87.1)	13 (12.9)	15.93	
Medullary	23 (92.0)	2 (8.0)	(0.01)	**0.03**
Miscellaneous	7 (77.8)	2 (22.2)		
Special type	41 (78.8)	11 (21.2)		

Bold indicates the significant values

### SLC7A8 expression in molecular BC subtypes

High expression of *SLC7A8* mRNA was significantly associated with hormone receptor positive (ER+ and PgR+) and HER2− BC (all *P* < 0.001, Table 2). Likewise, *SLC7A8* mRNA was highly expressed in non-triple negative (TN) compared with TN tumours (*P* < 0.001, Table 2). These results were validated using Breast Cancer Gene-Expression Miner (Supplementary Fig 2D–2G). Similarly, high SLC7A8 protein expression was associated with HER2 negative BC (*P* = 0.01) and although it was expressed primarily in hormone receptor positive tumours this did not reach significance (Table 2).

**Table 2:** Expression of SLC7A8 in breast cancer and the expression of other molecular biomarkers

	SLC7A8							
	mRNA				protein			
	Low *n* (%)	High *n* (%)	*χ*^2^ (*P* value)	Adjusted *P* value	Low *n* (%)	High *n* (%)	*χ*^2^ (*P* value)	Adjusted *P* value
ER								
Negative	365 (77.2)	108 (22.8)	553.2 (2.5 × 10^–122^)	** < 0.0001**	296 (89.4)	35 (10.6)	0.09 (0.76)	1.52
Positive	283 (18.9)	1215 (81.1)			1083 (88.8)	136 (11.2)		
PR								
Negative	453 (48.3)	484 (51.7)	193.6 (5.0 × 10^–44^)	** < 0.0001**	559 (91.0)	55 (9.0)	3.53 (0.06)	0.30
Positive	195 (18.9)	839 (81.1)			807 (88.0)	110 (12.0)		
HER2								
Negative	519 (30.1)	1206 (69.9)	48.7 (2.9 × 10^–12^)	** < 0.0001**	1174 (88.3)	155 (11.7)	8.82 (0.003)	**0.01**
Positive	129 (52.4)	117 (47.6)			198 (95.2)	10 (4.8)		
Triple negative								
No	388 (23.5)	1263 (76.5)	405.07 (4.3 × 10^–90^)	** < 0.0001**	1169 (89.4)	139 (10.6)	0.56 (0.45)	1.80
Yes	260 (81.3)	60 (18.8)			207 (87.7)	29 (12.3)		
*TP53* mutations								
Wild-type	204 (28.5)	512 (71.5)	38.36 (4.6 × 10^–9^)	** < 0.0001**	Not available			
Mutation	59 (59.6)	40 (40.4)						
p53 protein								
Negative	Not available				860 (89.0)	106 (11.0)	0.24 (0.62)	1.86
Positive					435 (89.9)	49 (10.1)		

Bold indicates the significant values

Regarding the association of *SLC7A8* CN and mRNA with the intrinsic (PAM50) subtypes, *SLC7A8* CN gain was mainly observed in luminal B tumours (*P* < 0.001, Supplementary Table 2), whereas high mRNA expression was observed primarily in luminal A and B tumours and to lesser extent in HER2+BC (*P* < 0.001, Fig. 1f). In the METABRIC Integrative Clusters, high *SLC7A8* mRNA expression was associated with clusters 7 and 8 which embrace ER+ tumours predominately of the luminal A intrinsic subtype (*P* < 0.001, Fig. 1g). Similar associations of *SLC7A8* mRNA with the molecular subtypes were seen using Breast Cancer Gene-Expression Miner (Supplementary Fig. 2H).

Although the result of the association of SLC7A8 protein in the defined IHC subtypes were not nominally significant, it also showed higher SLC7A8 expression in the ER+ low proliferation tumours compared with the other subtypes (Table 1).

## SLC7A8 expression and other associated markers

The correlations of *SLC7A8* mRNA with other relevant genes were investigated using the METABRIC dataset (Table 3). These genes were selected based on previous publications showing a functional association between *SLC7A8* and glutamine transport or metabolism [6, 26–28]. High *SLC7A8* mRNA expression was significantly associated with enzymes involved in glutamine metabolism: glutaminase (*GLS* and *GLS2;P* < 0.001), which mediate the conversion of glutamine to glutamate. While the correlation with *GLS* was negative, it was positive with *GLS2*. There was also a positive association with the enzymes which mediate conversion of glutamine to proline, namely, *ALDH4A1* and *PRODH* (*P* < 0.001). In contrast, some glutamine transporters were negatively correlated with *SLC7A8* expression (*P* ≤ 0.009), including SLC1A5, SLC7A5, SLC7A6, SLC7A7, and SLC38A3, while others showed a positive association, including SLC7A9, SLC38A1, SLC38A2, and SLC38A7 (*P* ≤ 0.003). The associations between *SLC7A8* and glutamine metabolic enzymes and transporters were primarily observed within luminal A tumours and to lesser extent in luminal B, HER2+ and TN subtypes. High *SLC7A8* mRNA expression was associated with tumours which showed wild-type TP53 expression (*P* < 0.001, Table 3).

**Table 3:** Correlation of *SLC7A8* expression with the expression of other related genes in the METABRIC data

	*SLC7A8* mRNA expression									
	All cases (*n* = 1980)		Luminal A (*n* = 368)		Luminal B (*n* = 367)	HER2+ (*n* = 110)		Triple negative (*n* = 150)		
	Correlation coefficient (*P* value)					Adjusted *P* value				
Glutamine metabolism										
GLS	− 0.10 (0.000009)	**0.0001**	0.07 (0.05)	0.40	0.05 (0.19)	1.89	0.01 (0.85)	3.84	− 0.09 (0.08)	1.52
GLS2	0.25 (1.1 × 10^–30^)	** < 0.0001**	0.07 (0.04)	0.36	0.01 (0.69)	2.69	0.28 (0.000006)	**0.0001**	− 0.03 (0.53)	1.06
ALDH4A1	0.22 (3.1 × 10^–23^)	** < 0.0001**	0.11 (0.004)	0.05	0.07 (0.09)	1.08	0.13 (0.03)	0.75	0.37 (3.1 × 10^–12^)	** < 0.0001**
PRODH	0.10 (0.000003)	**0.0001**	0.16 (0.00001)	**0.0002**	0.12 (0.005)	0.09	− 0.03 (0.56)	4.96	0.13 (0.01)	0.19
PYCR1	− 0.05 (0.01)	0.08	0.09 (0.01)	0.11	0.10 (0.02)	0.32	− 0.06 (0.33)	4.90	− 0.11 (0.05)	0.55
ALDH18A1	− 0.004 (0.86)	1.72	0.11 (0.003)	**0.04**	0.11 (0.01)	0.34	0.09 (0.16)	3.12	− 0.15 (0.004)	0.15
GLUL	0.21 (3.8 × 10^–21^)	** < 0.0001**	− 0.11 (0.002)	**0.03**	− 0.02 (0.58)	3.25	0.01 (0.83)	4.25	0.38 (3 × 10^–13^)	** < 0.0001**
GLUD1	0.42 (1.7 × 10^–84^)	** < 0.0001**	0.26 (2.9 × 10^–13^)	** < 0.0001**	0.15 (0.001)	**0.02**	0.27 (0.00001)	**0.0002**	0.11 (0.05)	0.70
Glutamine/glutamate transporters										
SLC1A5	− 0.07 (0.001)	**0.009**	0.06 (0.09)	0.54	0.007 (0.88)	1.76	− 0.03 (0.62)	4.34	− 0.20 (0.0002)	**0.003**
SLC3A2	− 0.08 (0.0003)	**0.003**	0.01 (0.61)	1.83	0.08 (0.06)	1.17	− 0.21 (0.001)	**0.01**	− 0.05 (0.35)	2.04
SLC6A19	− 0.007 (0.74)	2.96	− 0.002 (0.95)	1.90	− 0.04 (0.37)	3.22	0.03 (0.62)	4.98	0.03 (0.51)	1.59
SLC7A5	− 0.47 (1.3 × 10^–112^)	** < 0.0001**	− 0.155 (0.00003)	**0.0005**	− 0.11 (0.02)	0.60	− 0.18 (0.004)	0.48	− 0.45 (3.3 × 10^–18^)	** < 0.0001**
SLC7A6	− 0.12 (2.6 × 10^–8^)	** < 0.0001**	0.07 (0.05)	0.35	0.07 (0.09)	1.54	− 0.001 (0.98)	1.98	0.10 (0.07)	0.72
SLC7A7	− 0.25 (5.9 × 10^–31^)	** < 0.0001**	− 0.21 (2.3 × 10^–8^)	** < 0.0001**	− 0.14 (0.001)	**0.02**	− 0.33 (7.8 × 10^–8^)	** < 0.0001**	0.20 (0.0002)	**0.005**
SLC7A9	0.21 (1.2 × 10^–20^)	** < 0.0001**	1.33 (0.0003)	**0.005**	0.15 (0.001)	**0.02**	0.35 (1.7 × 10^–8^)	** < 0.0001**	− 0.06 (0.25)	1.92
SLC38A1	0.25 (1.3 × 10^–29^)	** < 0.0001**	0.27 (8.4 × 10^–14^)	** < 0.0001**	0.22 (7.5 × 10^–7^)	** < 0.0001**	0.12 (0.05)	0.84	− 0.21 (0.00007)	**0.001**
SLC38A2	0.08 (0.0003)	**0.003**	0.12 (0.001)	**0.01**	0.02 (0.65)	2.76	− 0.003 (0.96)	2.94	0.13 (0.01)	0.13
SLC38A3	− 0.14 (3 × 10^–11^)	** < 0.0001**	0.05 (0.13)	0.65	0.09 (0.04)	0.84	0.19 (0.002)	0.06	− 0.19 (0.0003)	0.06
SLC38A5	0.02 (0.28)	1.68	0.10 (0.006)	0.07	0.03 (0.46)	3.00	0.11 (0.06)	2.08	0.13 (0.01)	0.60
SLC38A7	0.08 (0.0001)	**0.001**	0.22 (5.9 × 10^–10^)	** < 0.0001**	0.14 (0.002)	**0.03**	0.31 (7.1 × 10^–7^)	** < 0.0001**	0.26 (9.6 × 10^–7^)	** < 0.0001**
SLC38A8	− 0.05 (0.02)	0.14	− 0.07 (0.03)	0.30	− 0.06 (0.14)	1.90	− 0.07 (0.26)	3.63	0.07 (0.19)	1.75
SLC7A11	− 0.007 (0.75)	2.25	− 0.04 (0.22)	0.88	0.05 (0.21)	2.96	0.04 (0.44)	5.04	0.05 (0.32)	1.75

Bold indicates the significant values

SLC7A8 protein was significantly expressed with high GLS and GLS2 enzymes (*P* < 0.001 and *P* = 0.006, Table 4), respectively. High PRODH, ALDH18A1, and ALDH4A1 were expressed in breast tumours with high SLC7A8 expression (all *P* ≤ 0.004, Table 4). High SLC7A8 expression was associated with low levels of SLC7A5 (*P* < 0.03, Table 4), whereas paradoxical associations were observed with the other transporters, SLC38A2 and SLC7A11 (*P* ≤ 0.02, Table 4).

**Table 4:** Association between SLC7A8 protein expression and other biomarkers

	SlC7A8 protein		
	Low, *n* (%)	High, *n* (%)	*χ*^2^ (*P* value)
PRODH			
Negative	255 (89.8)	29 (10.2)	10.93 (0.001)
Positive	46 (74.2)	16 (25.8)	
GLS			
Negative	226 (92.6)	18 (7.4)	18.45 (0.00001)
Positive	118 (77.6)	34 (22.4)	
GLS2			
Negative	171 (89.5)	20 (10.5)	7.41 (0.006)
Positive	155 (79.5)	40 (20.5)	
ALDH18A1			
Negative	190 (91.8)	17 (8.2)	8.17 (0.004)
Positive	157 (82.2)	34 (17.8)	
ALDH4A1			
Negative	185 (92.0)	16 (8.0)	11.19 (0.001)
Positive	152 (80.4)	37 (19.6)	
Positive			
SLC7A5			
Negative	1040 (88.1)	140 (11.9)	4.66 (0.03)
Positive	253 (92.7)	20 (7.3)	
SLC38A2			
Negative	992 (89.5)	116 (10.5)	10.21 (0.001)
Positive	89 (79.5)	23 (20.5)	
SLC7A11			
Negative	465 (92.1)	40 (7.9)	4.84 (0.02)
Positive	580 (88.1)	78 (11.9)

**Table 5:** SLC7A8 mRNA/ protein expression and patient outcome in the all breast cancer cases

Parameter	SLC7A8 mRNA		SLC7A8 protein	
	Hazard ratio (95% CI)	*P *value	Hazard ratio (95% CI)	*P* value
SLC7A8	0.70 (0.52–0.94)	**0.01**	0.57 (0.33–0.96)	**0.03**
Lymph node stage	2.00 (1.56–2.55)	**2.4 × 10**^**–8**^	1.89 (1.62–2.22)	**8.7 × 10**^**–16**^
Size	1.48 (0.99–2.20)	0.05	1.39 (1.08–1.78)	**0.009**
Grade	1.41 (1.08–1.82)	**0.01**	2.49 (1.97–3.16)	**2.8 × 10**^**–14**^

Bold indicates the significant values

### SLC7A8 expression and patient outcome

**Fig. 3:**
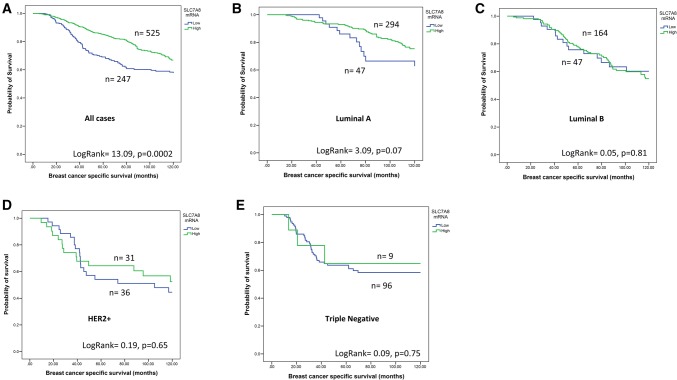
*SLC7A8* mRNA and breast cancer patient outcome. **a***SLC7A8* vs BCSS in all cases, **b***SLC7A8* vs BCSS in luminal A tumours, **c***SLC7A8* vs BCSS in Luminal B tumours, **d***SLC7A8* vs BCSS in HER2+ tumours, **e***SLC7A8* vs BCSS in Triple Negative tumours

**Fig. 4:**
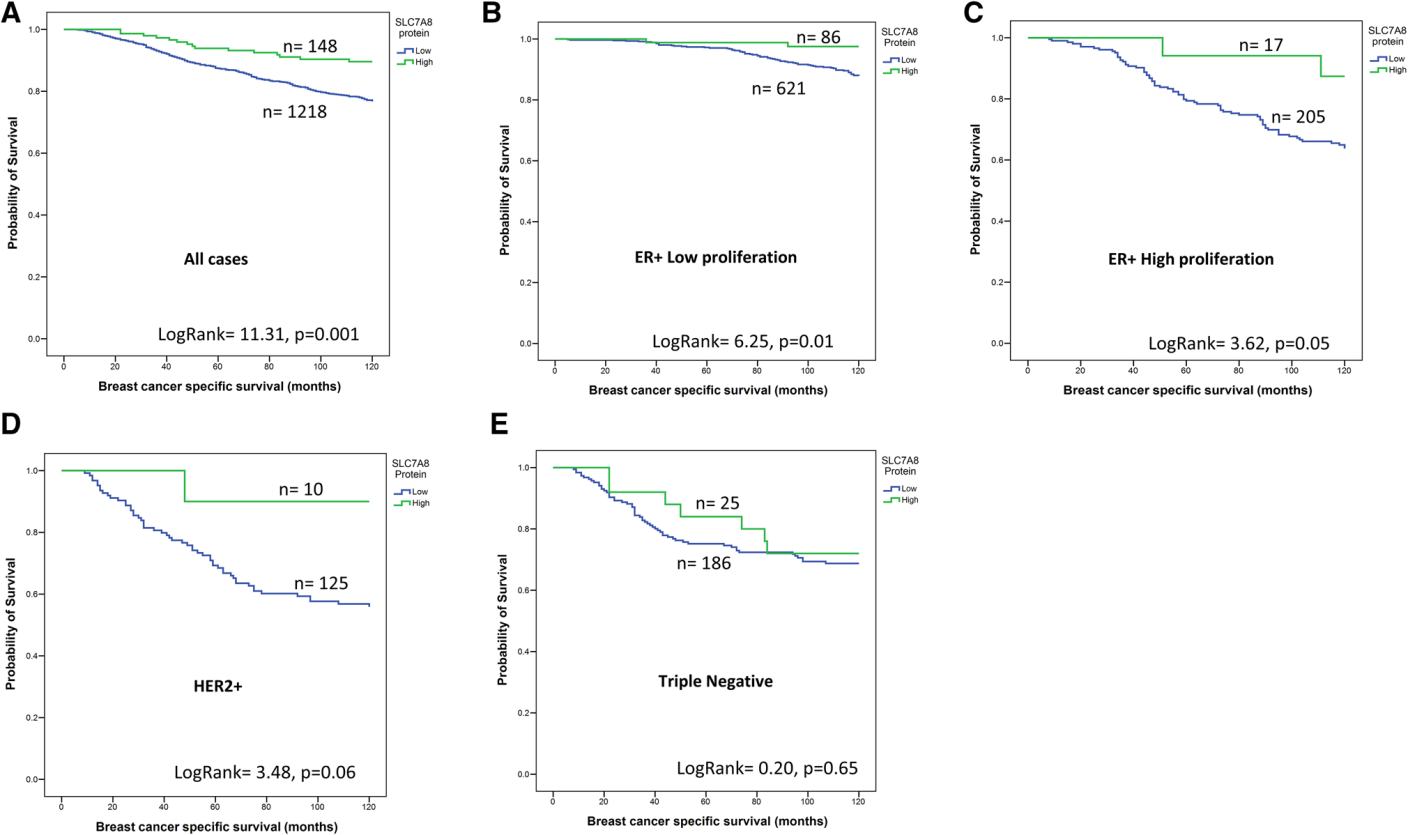
SLC7A8 protein and breast cancer patient outcome. **a** SLC7A8 vs BCSS in all cases, **b** SLC7A8 vs BCSS of ER+—low proliferation tumours, **c** SLC7A8 vs BCSS of ER+—high Proliferation tumours, **d** SLC7A8 vs BCSS of HER2+ tumours, **e** SLC7A8 vs BCSS of Triple Negative tumours

High expression of *SLC7A8* mRNA and protein was associated with longer BCSS (*P* ≤ 0.001, Figs. 3a and 4a). While *SLC7A8* mRNA expression was not predictive for BCSS in any specific molecular class (Fig. 3b–e), high expression of SLC7A8 protein was predictive of good survival in only the ER + low proliferation tumours (*P* = 0.01, Fig. 4b). There was no association between SLC7A8 protein and outcome in ER+ high proliferation, HER2+ or TN subtypes (Fig. 4c–e). Multivariate Cox regression analysis showed that *SLC7A8* mRNA and SLC7A8 protein were predictors of longer BCSS independent of tumour size, grade, and lymph node stage (*P* = 0.01 and *P* = 0.03, Table 5) respectively.

Likewise, high SLC7A8 protein expression was associated with longer distant metastases-free survival (DMFS) (*P* < 0.001, Supplementary Fig. 3A) within the ER+ low proliferation class (*P* = 0.003, Supplementary Fig. 3B) but not with other subtypes (Supplementary Fig. 3C, 2E). The relationship between high *SLC7A8* mRNA expression and good patient outcome was verified using Breast Cancer Gene-Expression Miner (Supplementary Fig. 4A, 4B).

## Discussion

BC represents a group of heterogeneous diseases that vary at the histopathological and molecular levels. These subtypes differ in their biology, clinical outcome, and response to therapy [29]. In addition, different BC subtypes showed disparity in their metabolic profiles and nutritional requirements. ER+/luminal tumours are the predominant BC subtype [30, 31] and characterised by having better prognosis and lower mortality rates as well as being targets for endocrine therapy [32]. In clinical practice, however, recognising patients who are likely to exhibit relapse or distant metastasis is challenging. Therefore, understanding the biology of BC is crucial in the pursuit of identifying targets for treatment and/or prognosis of BC patients particularly those with luminal tumours.

Altered metabolic pathways in human cancers are imperative to support cell proliferation and survival. Amino acid metabolism can vary substantially among BC subtypes, where TNBC display increased activity of amino acid consumption and metabolism compared with ER+ tumours [33, 34], suggesting that the latter subtype may allow for expression of lower levels of amino acid metabolic markers or express solute carriers that have lower affinity for their substrates. This study has revealed, for the first time, that SLC7A8 is a key amino acid transporter in the most predominant low proliferative ER+ tumours.

Unlike SLC7A5, SLC7A8 lacks studies that illustrate its prognostic role in human cancer. Data from Oncomine revealed a significant upregulation of SLC7A8 in several cancers, including breast, colorectal, head and neck, leukaemia, lymphoma, and melanoma [35]. However, this only has been validated at the mRNA level in a subset of breast tumours [15] and melanoma cell lines, which showed however more than five times increase in *SLC7A5* expression compared to *SLC7A8* [36]. Herein, we used large BC cohorts to reveal the significant associations between the high SLC7A8 expression, at mRNA and protein levels, and good prognostic clinicopathological parameters, including small tumour size, low tumour grade, and good NPI.

With respect to BC subtypes, the lowest levels of *SLC7A8* mRNA were observed in the ER- and TNBC tumours, while *SLC7A8* was higher in the ER+ subtypes which was more prominent in the luminal A tumours. These results were compatible with other studies which showed that *SLC7A8* mRNA was expressed in the ER+, MCF7, cell line but not in the ER−, MDA-MB-231 cells [37]. Furthermore, we have shown that SLC7A8 was associated with better patient outcome and longer DMFS in the ER+ low proliferative tumours only and not in the other subtypes. Thakkar et al. also found that upregulation of *SLC7A8* alongside *GATA3* and *MLPH* significantly associated with longer relapse free survival in ER+ lymph node positive breast tumours [15]. These results may suggest that ER+ low proliferative tumours settle for the lower affinity transporter, SLC7A8, to satisfy their nutritional needs as they exhibit lower metabolic activity compared to the aggressive forms of BC.

The relationship between hormone receptor positivity and *SLC7A8* indicate that hormone receptors have a possible role in stimulating *SLC7A8* expression. It has been shown that 17β estradiol, in ER+ BC cells, regulates l-leucine uptake through SLC7A5 and SLC7A8 while no effect was observed in the ER- BC cells [4]. It has been further shown that SLC7A8 has oestrogen-dependent expression, in ER+ BC cells, and the existence of inhibitors of oestrogen signalling pathway (ICI182780 and tamoxifen) eliminates the oestrogen-induced upregulation of *SLC7A8* [15]. Another study also revealed that progesterone significantly upregulates *SLC7A8* mRNA and SLC7A8 protein expressions, in uterine leiomyoma tissues, and knockdown of *SLC7A8* markedly increased leiomyoma cell proliferation [16].

*P53* is a well-known tumour suppressor gene that responds to various stress signals through modulating other cellular processes, including cell cycle arrest and apoptosis. *P53* also has a role in mediating other cellular mechanisms such as regulating metabolic pathways, including glutamine metabolism by inducing *GLS2* expression [38]. Interestingly, this study showed a positive association between wild-type *P53* and *SLC7A8.* This suggests that SLC7A8 may contribute to P53-dependent tumour suppression, which resulted in the presence of favourable prognosis and patient outcome in tumours expressing high SLC7A8. In addition, *SLC7A8* was upregulated alongside two tumour suppressor genes, CEACAM1 and BMP2, in human colon cancer cells after exposure to anti-tumour agent [39].

We have previously showed that SLC7A5 is highly expressed in the aggressive BC subtypes and it was predictive of poor prognosis and poor patient outcome [14] while analysing SLC7A8 in the same cohort has resulted in opposite findings. Furthermore, this study showed that SLC7A8 at both mRNA and protein levels were mutually exclusive with SLC7A5 expression. It is noteworthy that although SLC7A5 and SLC7A8 have similar substrate selectivity and function, the latter has narrower tissue expression pattern and exhibits lower affinity to its substrates [6]. There is also evidence which suggests that the role of SLC7A8 is limited to equilibration of amino acids distribution across the cell membrane while SLC7A5 mediates the actual net of amino acid flux [40], which is required for further mTORC1 activation and cellular proliferation.

Furthermore, it seems that both solute carriers have contrasting effect in tumourigenesis and they could be a subject of different regulatory mechanisms, as *SLC7A5* expression is induced by c-MYC while hormone receptors appear to control *SLC7A8* expression [4, 14, 16].

This study further investigated the association of SLC7A8 expression with other solute carriers which involved in amino acid transport. While the majority of these transporters were negatively associated with SLC7A8, others were positively correlated, including SLC38A7 which showed consistent correlation in all BC subtypes. Positive associations with several glutamine metabolic enzymes were also detected, among these, GLS2 and glutamate dehydrogenase (GLUD1) which are associated with tumours of good prognosis and favourable patient outcome [21, 41]. Some variability in the expression of investigated markers across molecular subtypes was observed. For example, luminal A tumours were the main class which showed association between *SLC7A8* and markers required for glutamine transport and metabolism. This could be attributed to the increased SLC7A8 expression and function in this particular BC subtype.

It is noteworthy that different transcriptional pathways such as c-MYC oncogenic transcription, hormone receptors and nutrient starvation responses regulate the expression of amino acid transporters in human cancers [27, 35]. This also applies to SLC7A5 and SLC7A8, as both belong to the system L transport family but their expression is controlled by different pathways. Furthermore, both solute carriers mediate the uptake of large neutral amino acids, with the latter showing decreased transporting capacity. However, SLC7A8 can also accept smaller neutral amino acids, such as glycine, alanine, serine, cysteine, and glutamine [42], which are known substrates of the key system A amino acid transporter, SLC1A5 that is highly expressed in TNBC [6, 43]. These statements indicate that SLC7A8 could be a key transporter in luminal A tumours through determining the actual net flux of not only the large but also the small neutral amino acids.

## Conclusion

This study has revealed that the solute carrier SLC7A8 is an independent good prognostic marker in BC. Overexpression of SLC7A8 appears to have tumour suppressive characteristics especially in the low proliferative ER+ subtype, thus it could act as a potential prognostic factor. Functional assessment is necessary to reveal the specific role played by this amino acid transporter in the low proliferative ER+ tumours.

## Electronic supplementary material

Below is the link to the electronic supplementary material.

Supplementary file1 (DOCX 1064 kb)

## Data Availability

The dataset analysed during the current study are available from the corresponding author on reasonable request.
