# Supplementary material for: The solute carrier SLC7A8 is a marker of favourable prognosis in ER-positive low proliferative invasive breast cancer
Source: Breast Cancer Res Treat. 2020 Mar 21;181(1):1–12. doi: 10.1007/s10549-020-05586-6 (PMC7182634; doi:10.1007/s10549-020-05586-6)
Supplement: Supplementary file 1 — Supplementary file1 (DOCX 1064 kb) [file 10549_2020_5586_MOESM1_ESM.docx]

**Supplementary Table 1.** Clinicopathological parameters of the METABRIC and Nottingham breast cancer series.

|  | **METABRIC series**  **n (%)** | **Nottingham TMA series**  **n (%)** |
| --- | --- | --- |
| **Age** |  |  |
| ≥ 50 years | 1426 (78.6) | 1784 (67.4) |
| ˂ 50 years | 424 (21.4) | 864 (32.6) |
| **Tumour size** |  |  |
| ≥ 2cm | 1337 (68.2) | 1176 (44.4) |
| ˂2cm | 623 (31.8) | 1471 (55.6) |
| **Grade** |  |  |
| 1 | 169 (9.0) | 421 (15.9) |
| 2 | 770 (40.7) | 988 (37.4) |
| 3 | 952 (50.3) | 1235 (46.7) |
| **Tumour type** |  |  |
| Ductal (including mixed) | 1545 (83.6) | 2257 (85.3) |
| Lobular | 148 (8.0) | 221 (8.4) |
| Medullary-like | 32 (1.7) | 39 (1.5) |
| Miscellaneous | 12 (0.6) | 16 (0.6) |
| Special type | 113 (6.1) | 113 (4.2) |
| **Vascular Invasion** |  |  |
| Definite | Not available | 833 (31.5) |
| Negative/Probable |  | 1808 (68.5) |
| **Lymph Node Stage** | | |
| 1 | 1035 (52.5) | 674 (61.0) |
| 2 | 623 (31.5) | 341 (30.8) |
| 3 | 315 (16.0) | 91 (8.2) |
| **Follow-up Status** |  |  |
| Alive | 1070 (55.7) | 1679 (63.4) |
| Died from Breast Cancer | 505 (26.3) | 647 (24.4) |
| Died from other causes | 345 (18.0) | 321 (12.2) |
| **ER** |  |  |
| Negative | 472 (23.8) | 570 (21.6) |
| Positive | 1508 (76.2) | 2067 (78.4) |
| **PgR** |  |  |
| Negative | 938 (47.4) | 1047 (41.4) |
| Positive | 1042 (52.6) | 1483 (58.6) |
| **HER2** |  |  |
| Negative | 1734 (87.5) | 2297 (88.5) |
| Positive | 246 (12.5) | 298(11.5) |

**Supplementary Table 2**. Copy number aberrations of *SLC7A8* in METABRIC breast cancer series and their associations with *TP53* gain and breast cancer subtypes.

| **SLC7A8 copy number** | | | | | | |
| --- | --- | --- | --- | --- | --- | --- |
| **Gain** | | | | **Loss** | | |
|  | **No**  **n(%)** | **Yes**  **n(%)** | **χ2**  **(p-value)** | **No**  **n(%)** | **Yes**  **n(%)** | **χ2**  **(p-value)** |
| **PAM50 subtypes** | | | | | | |
| Luminal A | 700 (97.5) | 18 (2.5) | 26.94  **(0.00002)** | 705 (98.2) | 13 (1.8) | 7.68  (0.10) |
| Luminal B | 448 (91.8) | 40 (8.2) |  | 477 (97.7) | 11 (2.3) |  |
| Basal | 314 (95.4) | 15 (4.6) |  | 315 (95.7) | 14 (4.3) |  |
| HER2+ | 226 (94.2) | 14 (5.8) |  | 237 (98.8) | 3 (1.3) |  |
| Normal-like | 196 (98.5) | 3 (1.5) |  | 195 (98.0) | 4 (2.0) |  |
| ***TP53* Gain** | | | | | | |
| No | 1878 (95.7) | 84 (4.3) | 34.69  **(3.58x10^-9^)** | 1917 (97.7) | 45 (2.3) | 0.42  (0.51) |
| Yes | 12 (66.7) | 6 (33.3) |  | 18 (100.0) | 0 (0.0) |  |


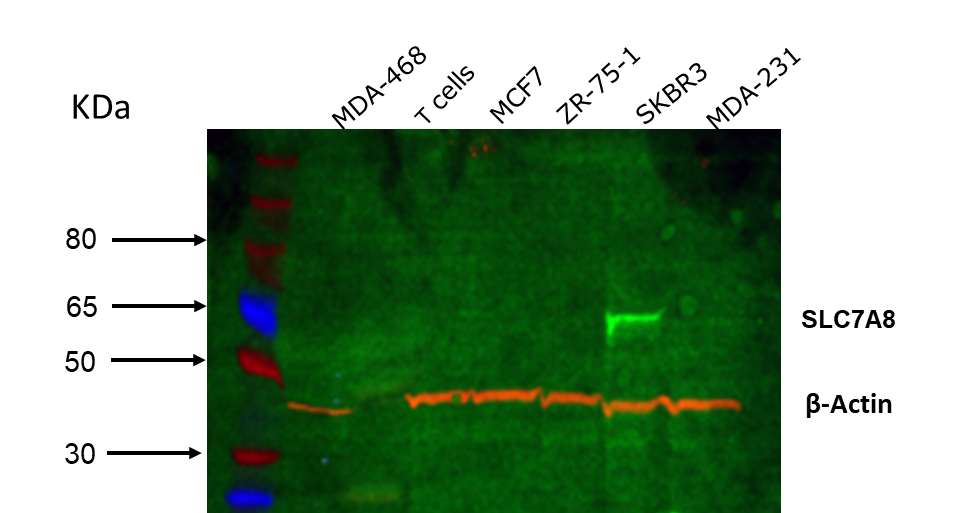


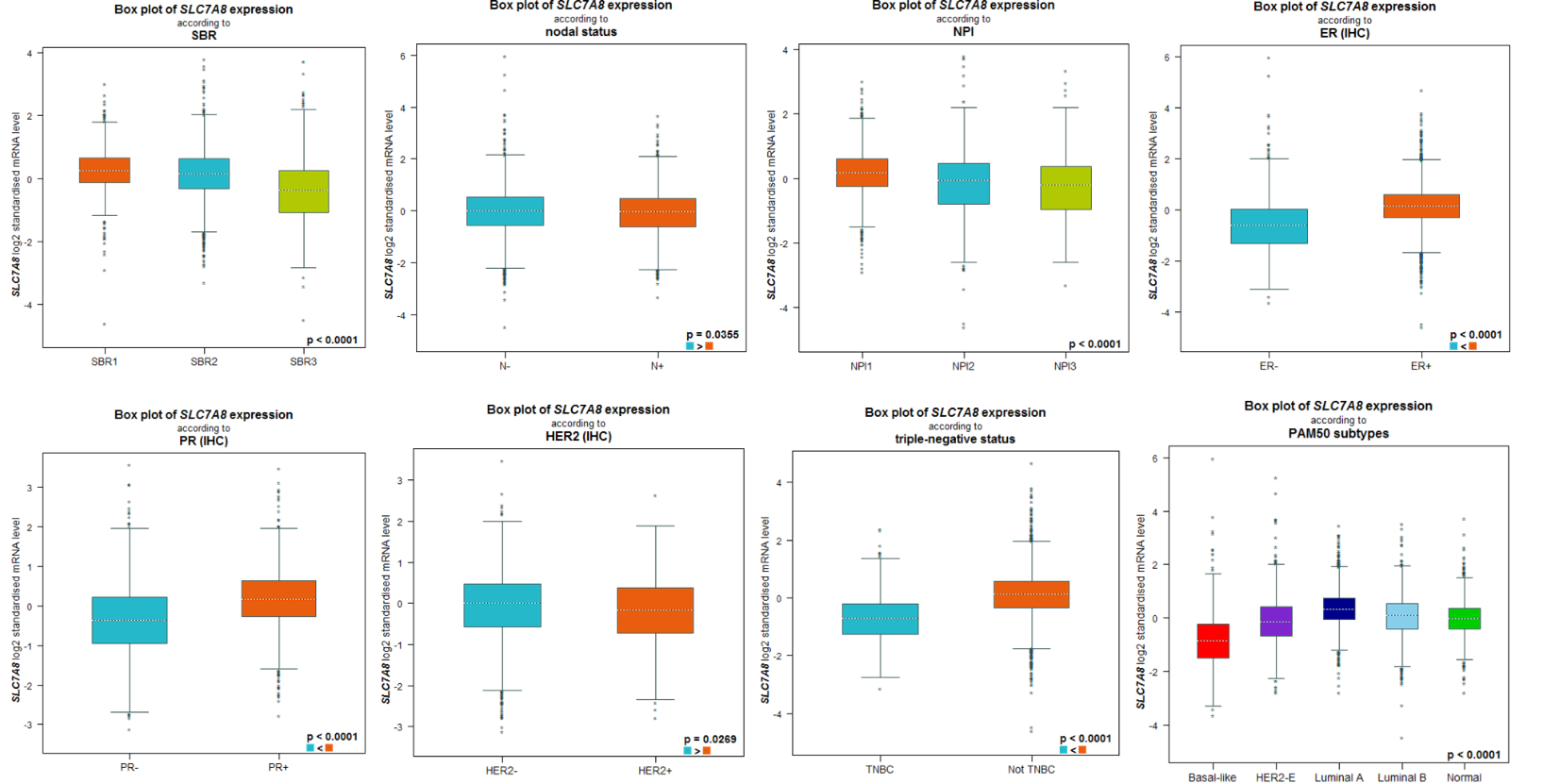
**Supplementary Figure 1**: Western blotting image for SLC7A8 validation. Expression was observed in SKBR3 breast cancer cell lysates at the expected molecular size.

**Supplementary Figure 2**: *SLC7A8* gene expression and its association, using Breast Cancer Gene-Expression Miner v4.0, with: A) tumour grade, B) Lymph node stage, C) NPI, D) ER status, E) PR status, F) HER2 status, G) Triple Negative status, H) PAM50 subtypes.


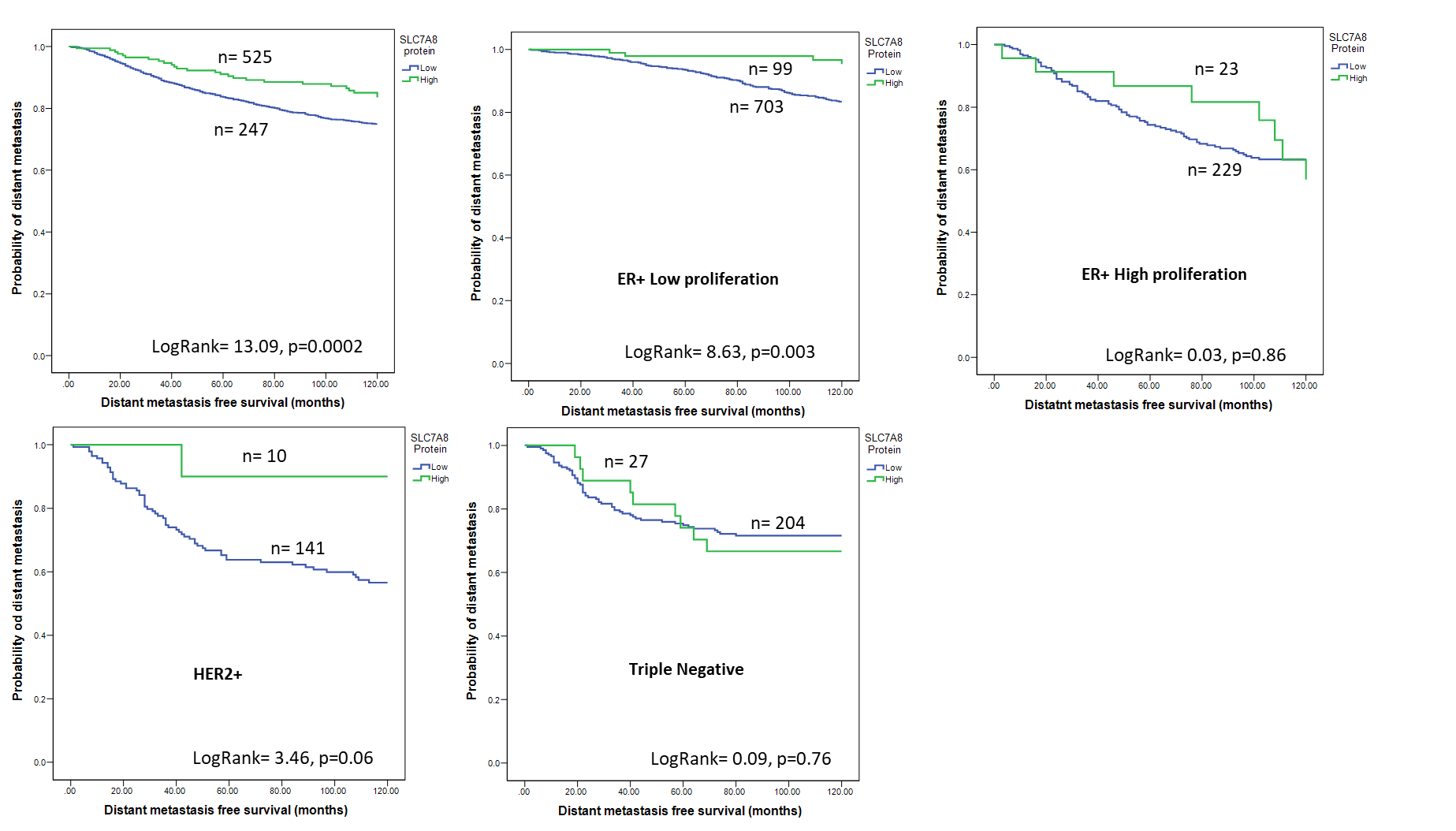


**Supplementary Figure 3.** SLC7A8 vs DMFS in A) all cases, B) ER+- Low Proliferation tumours, C) ER+- High Proliferation tumours, D) HER2+ tumours, E) Triple Negative tumours.


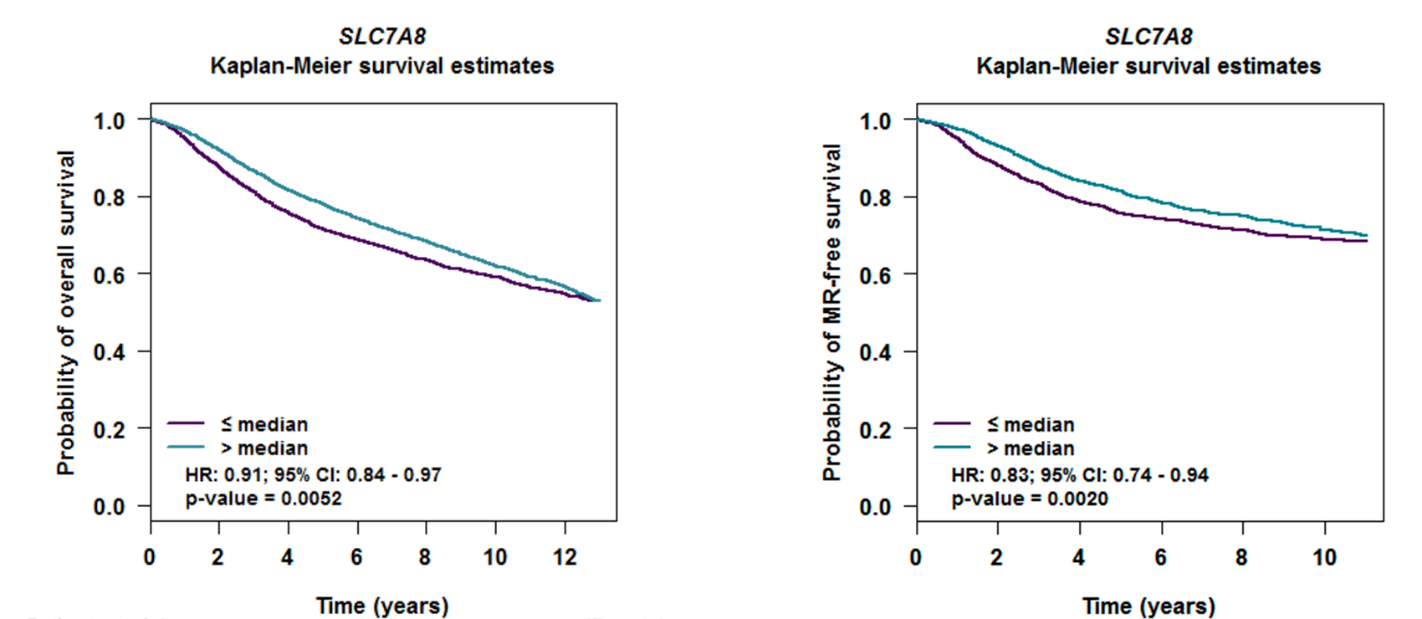


**Supplementary Figure 4:** *SLC7A8* mRNA and breast cancer patient outcome using Breast Cancer Gene-Expression Miner: A) Overall survival, B) metastatic relapse.
